# The (Non)Effects of Lethal Population Control on the Diet of Australian Dingoes

**DOI:** 10.1371/journal.pone.0108251

**Published:** 2014-09-22

**Authors:** Benjamin L. Allen, Luke K.-P. Leung

**Affiliations:** School of Agriculture and Food Sciences, the University of Queensland, Gatton, Queensland, Australia; Michigan Technological University, United States of America

## Abstract

Top-predators contribute to ecosystem resilience, yet individuals or populations are often subject to lethal control to protect livestock, managed game or humans from predation. Such management actions sometimes attract concern that lethal control might affect top-predator function in ways ultimately detrimental to biodiversity conservation. The primary function of a predator is predation, which is often investigated by assessing their diet. We therefore use data on prey remains found in 4,298 Australian dingo scats systematically collected from three arid sites over a four year period to experimentally assess the effects of repeated broad-scale poison-baiting programs on dingo diet. Indices of dingo dietary diversity and similarity were either identical or near-identical in baited and adjacent unbaited treatment areas in each case, demonstrating no control-induced change to dingo diets. Associated studies on dingoes' movement behaviour and interactions with sympatric mesopredators were similarly unaffected by poison-baiting. These results indicate that mid-sized top-predators with flexible and generalist diets (such as dingoes) may be resilient to ongoing and moderate levels of population control without substantial alteration of their diets and other related aspects of their ecological function.

## Introduction

Terrestrial carnivores face energetic constraints that influence many aspects their breeding, feeding and social ecology [1], [2]. Consequently, carnivores exhibit a wide variety of reproductive and hunting strategies to meet their energetic requirements. Smaller carnivores are often highly fecund, breeding continually throughout the year and adopting generalist diets primarily comprising of invertebrates and small mammals. Such carnivores might be typified by European badgers (*Meles meles*) or feral cats (*Felis catus*) (e.g. [3], [4]). Larger carnivores often breed only once annually, produce fewer young and rely on a much more narrow range of large mammal prey. Polar bears (*Ursus maritimus*), lions (*Panthera leo*) and grey wolves (*Canis lupus*) exhibit these characteristics (e.g. [5], [6]). The prey size preferred by carnivores scales positively with carnivore body mass, where a transition point from small to large prey preference occurs in carnivores weighing approximately 14–21 kg [1], [7]. Mid-sized carnivores in this weight range often exhibit hunting strategies that can reflect aspects of either larger or smaller carnivores, which can be modulated by various social constraints. These consumptive and non-consumptive functional effects of carnivores can strongly influence the structure and resilience of food webs and indirectly enhance biodiversity conservation [8]–[10].

Cooperative group hunting facilitates the acquisition of sufficient prey or energy resources typically unattainable by individuals foraging alone, and is a feature common to many large carnivores. For example, African wild dogs (*Lycaon pictus*) in larger groups prefer prey weighing 120–140 kg, whereas, those in smaller groups prefer prey of similar weight to themselves (16–32 kg; [11]). Lions also hunt in groups, preferring prey weighing 190–550 kg [5]. Large ungulates are primary prey for group-hunting grey wolves [6], [12]. Individuals within such groups possess hunting skills that, when combined with those of their associates, allow the group to be successful in capturing and subduing the prey resources they individually require. This suggests that alterations to group composition (or demography) may affect hunting success, individual fitness and ultimately the persistence of the group or population and their ecological function [13], [14]. The lethal control of carnivores (often undertaken for the protection of livestock, managed game or humans) is one way that alters the composition of carnivore populations [15]. Lethal control includes shooting, trapping or poisoning in different parts of the world, and can target problem individuals or entire populations across large areas. That the removal of individuals from group-hunting large carnivores can sometimes influence the sustainability of their populations is well known (e.g. [16], [17]), but limited information exists on the effects of lethal control or social disruption on the diet or function of mid-sized carnivores with flexible and adaptable foraging abilities.

As a type of dog, dingoes (*Canis lupus dingo* and hybrids) are classic mesopredators [18], yet usurped their present top-predator status subsequent to their introduction to Australia about 5000 years ago, coincident with decline and extinction of the thylacine (*Thylacinus cynocephalus*) on the mainland. Dingoes typically weigh 12–17 kg (Table 1), require about 750 g/kg body weight in food per day with semi-frequent watering [19], and are the most closely related canid to grey wolves ([18], [20]; but see also [21]). Dingoes breed once annually (with a birth peak in winter) and can exist individually or in groups of over 20 (usually 4–10; [19], [22]). Dingoes have flexible and generalist diets, and populations are known to persist on a variety of prey from invertebrates to water buffalo (*Bubalus bubalis*) (e.g [23], [24]). Some populations sometimes use group hunting to capture relatively large kangaroos (*Macropus* spp.; Australia's largest native terrestrial mammals; females 18–40 kg, males 55–85 kg), while others live in groups yet forage individually on small mammals (<200 g) or European rabbits (*Oryctolagus cuniculus*; ∼1.5 kg) [23], [25], [26]. The distribution of dingoes presently extends across ∼85% of the continent and is naturally expanding back into the few remaining areas where they were formerly eradicated in the early 1900s [27]. The broad-scale distribution of 1080-poisoned baits (hereafter referred to as ‘baiting’) is common across much of Australia to protect commercial sheep (*Ovis aries*), goats (*Capra hircus*) and cattle (*Bos indicus*, *B. taurus* or their crosses) from dingo predation [28]. Due to rapid reinvasion, dingo abundance is typically resilient to contemporary baiting practices over time [28]–[30]. However, baiting has been predicted by some to alter dingo function independent of changes in dingo abundance, inclusive of changing their diet from a relatively narrow variety of large prey to a broader variety of small and often threatened prey (e.g. [31]–[33]).

**Table 1:** Mean body weights of Australian dingoes from 17 wild populations.

Location	Mean body weight (kg)	N =	Reference
Kumbarilla Forest	13.0	7	[70],
Central Australia	13.5	50	[20]
Sturt Stony Desert	13.5	2	B. Allen, unpublished data
Tanami Desert	13.7	143	[72]
NE NSW forests	14.1	100	G. Ballard, unpublished data
Taunton National Park	14.6	9	[70], [71]
Victorian highlands	15.1	28	[20]
Dunluce Station	15.8	5	[70], [71]
Kosciuszko National Park	15.8	23	[73]
Blue Mountains	16.0	47	[74]
Kakadu National Park	16.3	19	[20]
Fraser Island	16.7	147	L. Behrendorff, unpublished data
Strzelecki Desert	16.8	17	[19], [35]
Peri-urban areas of greater Brisbane	17.0	32	B. Allen, unpublished data
Charleville	17.7	6	[70], [71]
Idalia National Park	18.0	4	[70], [71]
Stratford	19.6	7	[70], [71]
***TOTAL***	***15.7***	***646***	

In this study, we experimentally test this hypothesis by evaluating the effects of repeated broad-scale poison baiting programs on dietary similarity and diversity of Australian dingo populations. In doing so, we illustrate how a social and mid-sized top-predator can respond to the repeated removal of some individuals and the disruption of social groups. The aim of this study was to determine whether or not dietary diversity and similarity were different between dingo populations exposed to lethal control and those that were left intact. The simple and common approach we use to compare dingo diets in baited and unbaited areas should be readily applicable to a wide range of other carnivores of various body sizes.

## Materials and Methods

### Study sites and design

The study was conducted on three large and privately-owned beef cattle stations in the arid zone of northern South Australia (NSA). Quinyambie Station is located in the sandy Strzelecki Desert (−30.871887, 140.970354), has a mean annual rainfall (MAR) of ∼160 mm, and is comprised of parallel sand dunes dominated by hopbush (*Dodonaea viscosa*), buckbush (*Salsola kali*), and a variety of grasses and burrs including kerosene grass (*Aristida* spp.) and copperburr (*Sclerolaena* spp.). Cordillo Downs Station is in the far northeast of South Australia around the Sturt Stony Desert (−26.706477, 140.625876), receives a MAR of ∼167 mm, and incorporates both large, irregular sand dunes and extensive stony gibber plains. These contain beefwood (*Grevillea striata*) and spinifex (*Triodia* spp.) on the dunes, Mitchell grass (*Astrebla* spp.) on the plains, and red gum (*Eucalyptus camaldulensis*) and mineritchie (*Acacia cyperophylla*) in the drainage lines. Todmorden Station is located on the edge of the Pedirka Desert in the central-north of the state (−27.139073, 134.756423), has a MAR of ∼180 mm, and is comprised of sandy deserts dominated by mulga (*Acacia aneura*) stands, with stony plain and drainage line vegetation similar to Cordillo Downs Station. Rainfall means were derived from long-term Bureau of Meteorology (www.bom.gov.au) data collected daily from nearby weather stations at Birdsville (since 1892), Frome Downs Station (since 1889), Hamilton Station (since 1884), Innamincka (since 1882), Macumba Station (since 1891) and Todmorden Station (since 1949). Information on South Australian floral species was obtained from Kutsche and Lay [34].

Each of the three stations were divided into paired dingo-baited and unbaited treatment areas (separated by a buffer zone>20 km at the closest point) as part of a larger manipulative experiment investigating the effects of dingo control on the ecology and management of dingoes and threatened fauna (e.g. [29], [30]). Treatments were considered independent given that GPS tracking of 18 dingoes (from both baited and unbaited areas) showed that scat collection sites were separated by at least 1–2 dingo home ranges, with dingoes exhibiting high site fidelity in both treatments (mean daily travel distance = 14.0 km, range = 7.8–18.8 km/day; N = 3340 dingo-days of monitoring data; from [19], [35]). Baiting programs at the sites typically occurred twice each year in the baited areas (in autumn and spring), and results from associated studies indicated that dingo population abundance trends at our study sites were resilient to baiting given the typical and variable levels of population reductions and/or increases experienced (Table 2) and rapid reinvasion of baited areas [29], [30]. In a regional context (see [36] for details), Todmorden is within the most intensively baited region in NSA, while Cordillo Downs is surrounded by a mosaic of baited and unbaited areas. Quinyambie is bordered by the dingo barrier fence which separates sheep grazing areas from cattle grazing areas [27], [37], and apart from regular baiting which occurs in some parts of a 30 km buffer zone along the fence, the treatment areas are surrounded by unbaited areas. Additional history of lethal dingo control in NSA and further detail on additional outcomes of baiting at the study sites are available elsewhere (e.g. [23], [29], [30], [35], [36], [38]).

**Table 2:** Short-term changes (mean days since baiting = 54) in dingo passive tracking index (PTI) values in response to 11 poison-baiting programs undertaken at the study sites between July 2008 and August 2011, showing the net baiting-induced reductions or increases in dingo activity (adapted from [29]).

Baiting program ID	Site	Post-control survey date	% PTI change in the baited area	% PTI change in the unbaited area	Net% change in PTI in the baited area
CD1	Cordillo Downs	22-Jul-09	93.8%	−21.1%	114.8%
CD2	Cordillo Downs	20-Jan-10	21.8%	89.4%	−67.6%
Q1	Quinyambie	08-Jul-08	44.4%	70.0%	−25.6%
Q2	Quinyambie	30-Sep-09	5.3%	−48.4%	53.7%
Q3	Quinyambie	29-Jun-11	67.7%	31.8%	35.8%
Q4	Quinyambie	03-Aug-11	59.9%	−56.8%	116.7%
T1	Todmorden	15-Jan-09	0.0%	−604.7%	604.7%
T2	Todmorden	27-Feb-09	−75.0%	−2650.0%	2575.0%
T3	Todmorden	22-Apr-09	50.2%	67.2%	−17.0%
T4	Todmorden	23-May-09	100.0%	90.7%	9.3%
T5	Todmorden	04-Feb-10	−100.0%	77.8%	−177.8%

Note: Positive values denote% reductions in dingo activity; negative values denote% increases in dingo activity. Reductions>100% indicate that dingoes present prior to baiting were removed, along with additional immigrating dingoes as well.

Although a variety of prey were present at each site, the relative abundance and availability of each prey species was not consistent between sites and varied throughout the study period in order to provide *in situ* assessments of changes in dingo diets in places with predominately small-, large- or mixed-sized prey. Thus, fauna assemblages were different between the three sites but similar between treatments at each site (Table 3; see also [30]), which were located on the same property. Most of the mammalian prey species identified in dingo scats from a given site were also present at each other site [23]. However, feral pigs (*Sus scrofa*) were found only on Cordillo Downs [39] and some small mammals identified in dingo scats have restricted distributions [40] and are likely rare or absent at some sites [41]. Kangaroos are uncommon at Quinyambie, relatively common at Cordillo Downs and abundant at Todmorden ([42]–[44]; but see [30]). Rabbits are abundant at Quinyambie and uncommon at the other sites. Further information on the distribution of native and introduced prey species can be found elsewhere (e.g. [39], [40], [45], [46]).

**Table 3:** Total number of prey tracks (footprints) observed during 32 standardised surveys undertaken at the study sites during the study period (see [30] for details).

Site (N surveys)	Treatment	Birds	Rabbits	Small mammals∧	Macropods	Pigs	Echidnas	Frogs	Hopping-mice	Reptiles*	Goannas (*Varanus* spp.)
Quinyambie (14)	B	1026	922	1942	12	ND	1	0	6105	213	2
	UB	948	1234	1554	1	ND	1	3	8883	190	0
Cordillo Downs (7)	B	134	16	593	25	7	ND	0	356	136	3
	UB	459	53	980	20	0	ND	37	130	252	11
Todmorden (11)	B	496	221	1366	89	NP	ND	8	393	377	18
	UB	470	9	1333	108	NP	ND	0	272	173	15

ND = Present, but not detected; NP = Not present;

^∧^All dasyurids and rodents except for hopping-mice (*Notomys *spp.).

*All reptiles except *Varanus* spp., mostly agamidae and scincidae.

### Dingo scat collection and analysis

Dingo scats were collected from within each treatment area during repeated visits to the sites between May 2008 and May 2012. Dingo scats were distinguished from those of other predators (such as feral cats and red foxes, *Vulpes vulpes*) based on their size, shape, smell and placement [47]. Scat collection occurred six times at Cordillo Downs, nine times at Todmorden and 14 times at Quinyambie during this period. Because of the high abundance (and thousands of available scats) of dingoes at Quinyambie [19], [36], scat collection was restricted to discrete, fenced (to exclude cattle) areas around five permanent artificial livestock watering points (two in unbaited areas, three in baited areas). At the other two sites, scats were collected from a wide variety of waterpoints, vehicle tracks, dry creek crossings, intersections and other focal locations where dingoes were expected to defecate more frequently. The same locations within each treatment were surveyed for scats at each repeated visit to each site. Thus, there was unequal sampling effort between treatments and sites, but there was equal sampling effort within treatments between surveys at each site [38].

Dingo scats collected were first sterilised and washed by a professional service provider who then searched each scat for the remains of prey fauna and other food items. Mammal species were identified from diagnostic characteristics of their hair (described in [48]). Results were reported at the genus level (or higher) where there was ambiguity over positive species-level identification. Non-mammal food items were categorised simply as birds, reptiles (inclusive of both smooth- and rough-scaled species, such as agamidae or scincidae), invertebrates or vegetation; these were only described to the species level opportunistically (by staff at the South Australian museum) according to the incidental presence of diagnostic bones and other features in the scat (such as teeth or scales). Results are expressed as the ‘percent occurrence in scats’ because many of the species detected in scats were relatively rare or uncommon [49]. Associated data on spatiotemporal variation in scat collection rates and the overall diet of dingoes at the sites are available in Allen [38] and Allen and Leung [23].

We made no attempt to compare or contrast dingo diets between sites, but were primarily concerned with dingo dietary diversity and similarity between treatments within a given site. Therefore, we used Brillouin's Index to quantify and compare the *diversity* of dingo diets between baited and unbaited areas at each site according to the equation:
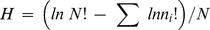
where *H* = diversity, *N* = total number of individual prey recorded and *n_i_* = number of individual prey items in the *i*th category [50]. Resulting *H* values typically range between 0 and 4.5, representing low and high dietary diversity, respectively. Similar values between treatments would indicate a similar range or suite of food and prey items is consumed by populations of dingoes in both baited and unbaited areas.

We also used Pianka's Index to quantify and compare the *similarity* between dingo diets in baited and unbaited areas at each site for each individual survey and overall (all surveys pooled) using the equation:
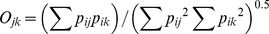
where *O* is the index of similarity or overlap, *j* and *k* are the dingo populations being compared (i.e. those in baited and unbaited areas), and *P_i_* is the frequency of occurrence of the *i*th prey or food type [51]. Using this technique, *O* values range between 0 and 1, where values of 0 indicate complete dissimilarity (i.e. no prey in common) and values of 1 indicate complete similarity (i.e. diets are identical). Generalized linear regression was used to assess whether or not diet similarity (the response variable) changed through time (the predictor variable). We considered using additional techniques to further explore dietary differences (such as those described in [52]), but given our results (see below) and the aims of our study (see above), we considered them to be unnecessary and/or inappropriate for our data. The two simple but robust techniques we use have been widely used by others to compare the diets of dingoes with those of other sympatric predators, where dietary overlap between sympatric predators typically ranges between *O* = 0.5–0.8 (e.g. [53]–[59]); *O* values exceeding 0.75 have been described as substantial, strong or significant dietary overlap in these studies.

All procedures were carried out under permit issued by the South Australian Department of Environment and Heritage's Wildlife Ethics Committee (WEC 16/2008).

## Results

**Figure 1:**
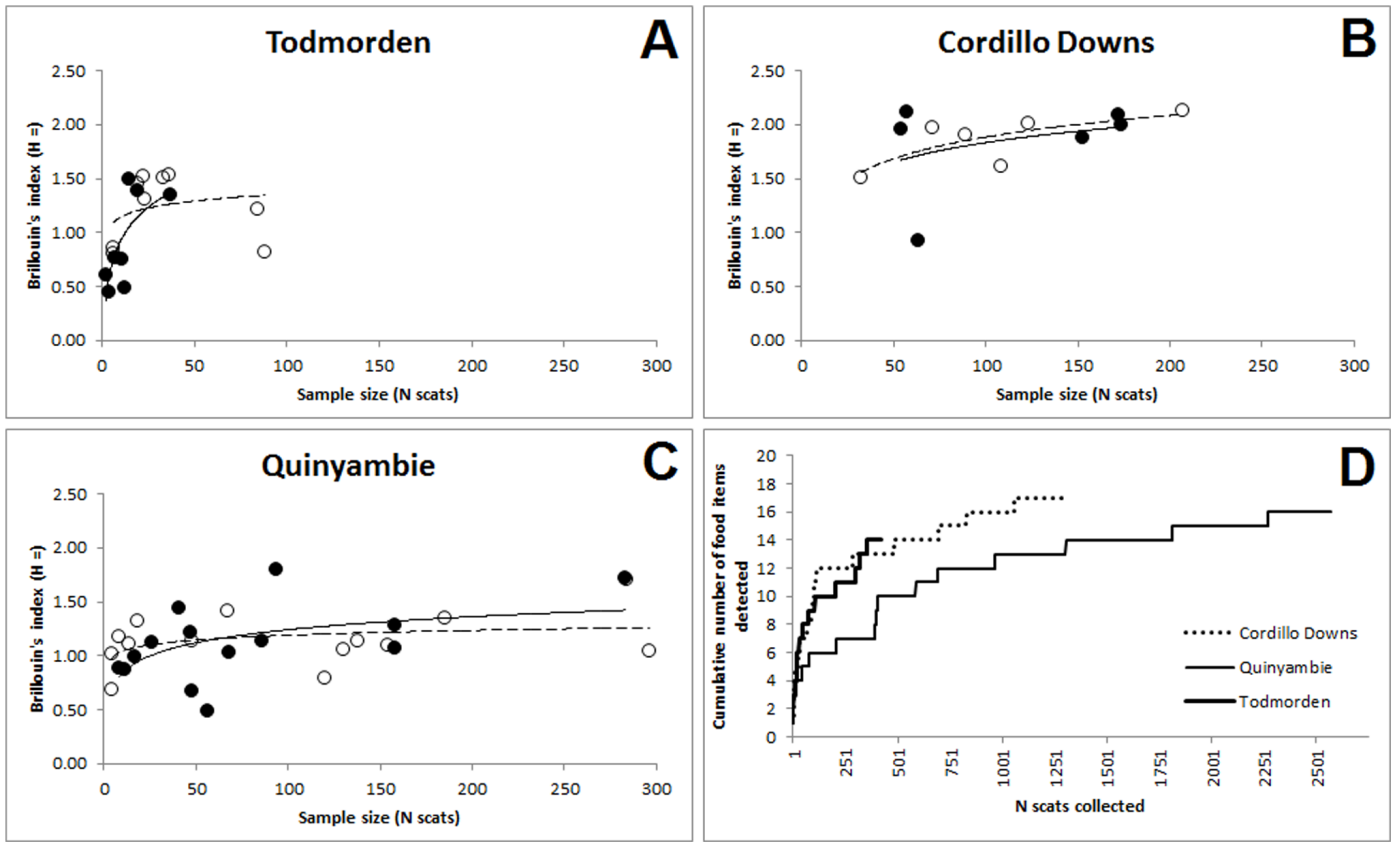
The relationship between Brillouin's index and sample size at (A) Todmorden, (B) Cordillo Downs and (C) Quinyambie, and the relationship between the number of dingo scat samples collected and the number of prey or food items detected in scats from each of these three sites in northern South Australia.

Baiting killed extant dingoes and temporarily reduced their population sizes at each site from time to time (Table 2), but baiting did not eliminate dingo populations, change the way dingoes interacted with sympatric predators, or change dingo movement behaviour and detectability [29], [35], [36]. We collected and analyzed 4,298 dingo scats – the second largest dingo diet study ever conducted [23], [38]. Analyses of the relationship between Brillouin's index values and sample size indicated that approximately 50 scats were required to reliably compare and contrast dingo diets at our sites (Fig. 1). A total of 1,881 scats were collected in baited areas and 2,417 in unbaited areas (Table 4). Between 107 and 1,470 scats were collected in a given treatment and site. Thus, our sample sizes were sufficient for our analyses. Previous studies of dingo diet have similarly determined that approximately 30 scats are required to reliably compare and contrast dingo diets at other sites (e.g. [58]).

**Table 4:** Sample sizes and Pianka's Index (*O* = ) values for dingo scats collected in baited and unbaited treatment areas during 29 surveys at three sites in northern South Australia, May 2008 to May 2012.

Study site	Survey Date		N scats		Pianka's Index
		Baited	Unbaited	Total	
Cordillo Downs	Oct-08	63	32	95	0.68
	Apr-09	54	89	143	0.98
	Jul-09	172	123	295	0.93
	Nov-09	174	207	381	0.91
	Jan-10	57	71	128	0.95
	Nov-10	153	108	261	0.89
	*Overall*	*673*	*630*	*1303*	*0.99*
Quinyambie	May-08	56	130	186	0.98
	Sep-08	48	154	202	0.99
	Mar-09	158	185	343	1.00
	Jun-09	158	296	454	0.99
	Sep-09	68	120	188	0.99
	Dec-09	283	138	421	0.99
	Jun-10	94	284	378	0.98
	Sep-10	41	48	89	1.00
	Apr-11	47	4	51	0.66
	Jun-11	11	8	19	0.68
	Jul-11	26	4	30	0.91
	Aug-11	8	14	22	0.96
	Feb-12	86	67	153	0.83
	May-12	17	18	35	0.78
	*Overall*	*1101*	*1470*	*2571*	*1.00*
Todmorden	Oct-08	11	6	17	0.95
	Jan-09	2	36	38	0.57
	May-09	37	84	121	0.98
	Aug-09	12	88	100	0.99
	Dec-09	0	33	33	N/A
	Feb-10	7	22	29	0.88
	Apr-10	4	6	10	0.76
	Sep-10	15	23	38	0.55
	Nov-10	19	19	38	0.89
	*Overall*	*107*	*317*	*424*	*0.96*

**Figure 2:**
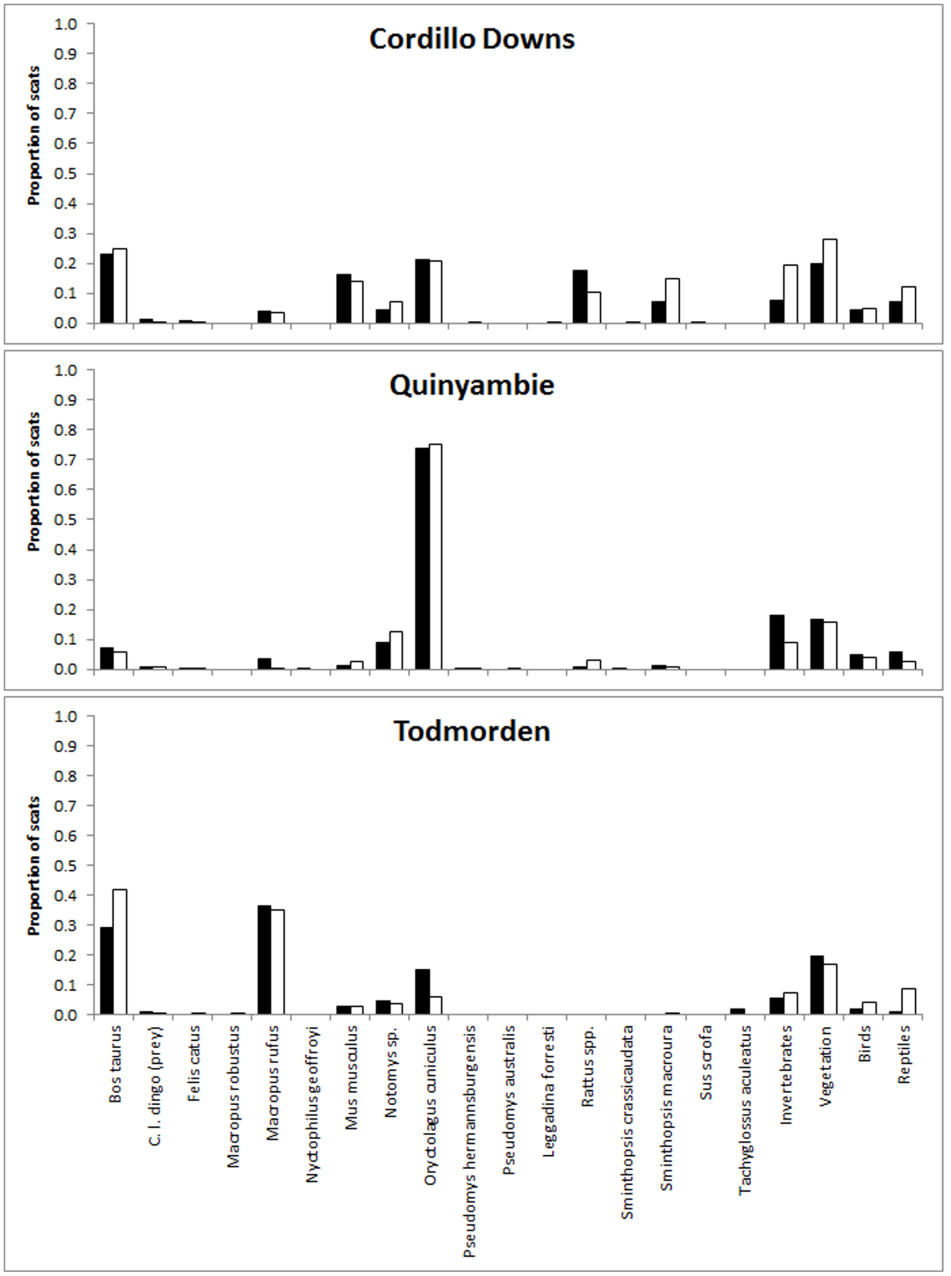
Proportion of prey and food items detected in dingo scats from baited (solid bars) and unbaited (hollow bars) treatment areas at three sites in northern South Australia, May 2008 to May 2012.

**Figure 3:**
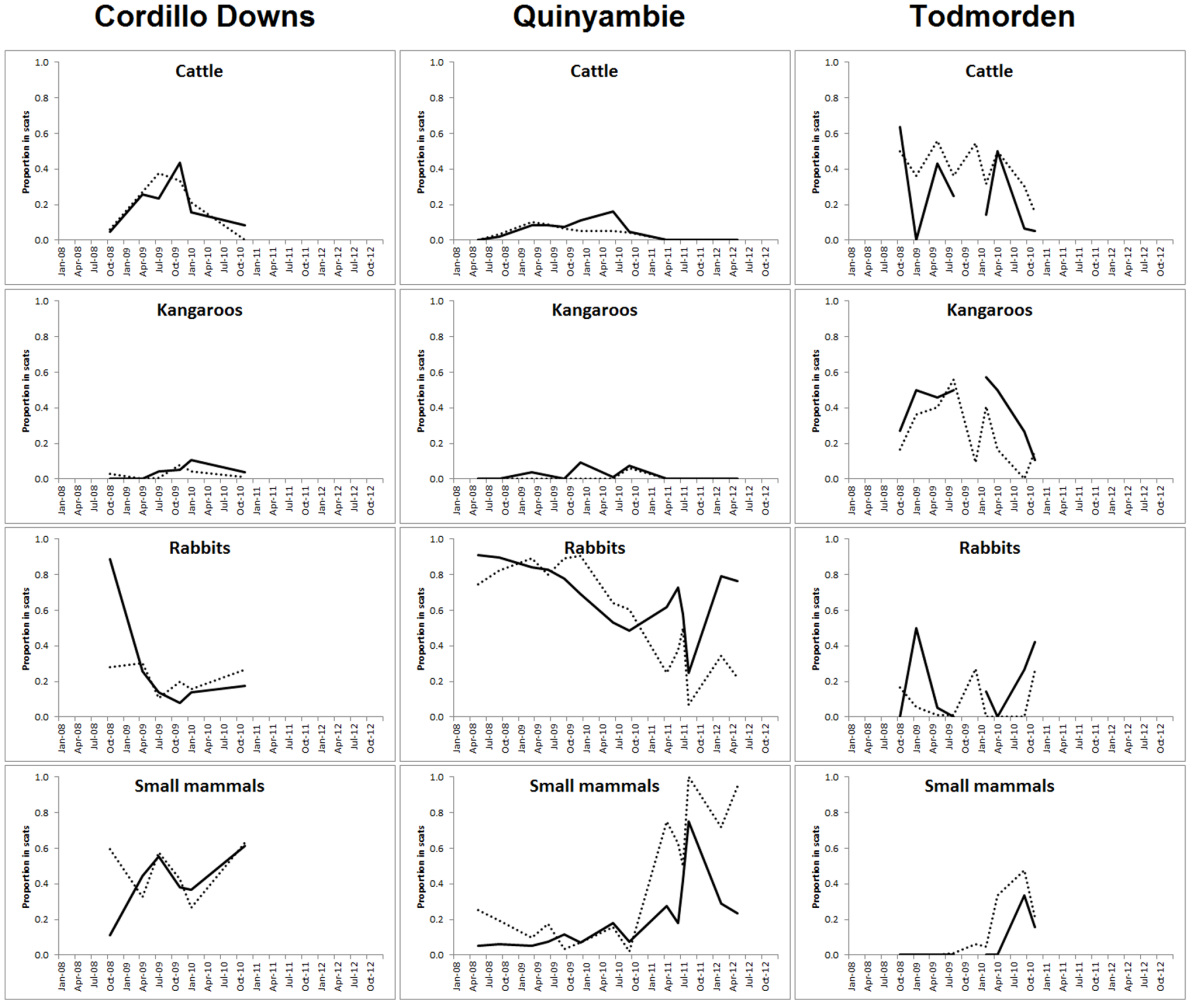
Trends in the proportion of cattle, kangaroo, rabbit and small mammal remains found in dingo scats in baited (solid lines) and unbaited (broken lines) treatment areas on Cordillo Downs (left), Quinyambie (centre) and Todmorden (right) in northern South Australia.

Between 14 and 17 different prey species or food items were detected in dingo scats at each site (Table 5, Fig. 1). The main prey consumed by dingoes were cattle, kangaroos, rabbits and a variety of small mammals, primarily dusky hopping-mice (*Notomys fuscus*), house mice (*Mus musculus*), stripe-faced dunnarts (*Sminthopsis macroura*) and long-haired rats (*Rattus villosissimus*) (Fig. 2; see also [23]). Vegetation and invertebrates also occurred relatively commonly in dingo diets. Temporal trends in the proportion of each of the main prey species in dingo scats were similar between treatments (Fig. 3). Brillouin's index (*H*) values for Cordillo Downs (baited = 2.24, unbaited = 2.25), Quinyambie (baited = 1.64, unbaited = 1.54) and Todmorden (baited = 1.70, unbaited = 1.79) indicated a near-identical diversity or suite of food items were consumed by dingoes between treatments at each site, with a greater diversity of items at Cordillo Downs than at the other two sites.

**Table 5:** The number (and proportion) of various food items found in 4,298 dingo scats collected from baited and unbaited treatment areas at three sites in northern South Australia, May 2008 to May 2012.

	Cordillo Downs	Cordillo Downs	Quinyambie	Quinyambie	Todmorden	Todmorden
Food item	Baited	Unbaited	Baited	Unbaited	Baited	Unbaited
N =	673	630	1101	1470	107	317
*Bos taurus*	155 (0.23)	156 (0.25)	80 (0.07)	82 (0.06)	31 (0.29)	133 (0.42)
*C. l. dingo* (prey)	7 (0.01)	1 (0.00)	6 (0.01)	12 (0.01)	1 (0.01)	2 (0.01)
*Felis catus*	4 (0.01)	1 (0.00)	1 (0.00)	4 (0.00)	0 (0.00)	1 (0.00)
*Macropus robustus*	0 (0.00)	0 (0.00)	0 (0.00)	0 (0.00)	0 (0.00)	2 (0.01)
*Macropus rufus*	28 (0.04)	22 (0.03)	39 (0.04)	4 (0.00)	39 (0.36)	111 (0.35)
*Nyctophilus geoffroyi*	0 (0.00)	0 (0.00)	1 (0.00)	0 (0.00)	0 (0.00)	0 (0.00)
*Mus musculus*	110 (0.16)	89 (0.14)	13 (0.01)	39 (0.03)	3 (0.03)	9 (0.03)
*Notomys* spp.	30 (0.04)	44 (0.07)	98 (0.09)	187 (0.13)	5 (0.05)	11 (0.03)
*Oryctolagus cuniculus*	143 (0.21)	130 (0.21)	811 (0.74)	1103 (0.75)	16 (0.15)	19 (0.06)
*Pseudomys hermannsburgensis*	0 (0.00)	1 (0.00)	1 (0.00)	1 (0.00)	0 (0.00)	0 (0.00)
*Pseudomys australis*	0 (0.00)	0 (0.00)	0 (0.00)	1 (0.00)	0 (0.00)	0 (0.00)
*Leggadina forresti*	0 (0.00)	1 (0.00)	0 (0.00)	0 (0.00)	0 (0.00)	0 (0.00)
*Rattus* spp.	118 (0.18)	65 (0.10)	7 (0.01)	43 (0.03)	0 (0.00)	0 (0.00)
*Sminthopsis crassicaudata*	0 (0.00)	1 (0.00)	2 (0.00)	0 (0.00)	0 (0.00)	0 (0.00)
*Sminthopsis macroura*	49 (0.07)	93 (0.15)	14 (0.01)	10 (0.01)	0 (0.00)	1 (0.00)
*Sus scrofa*	3 (0.00)	0 (0.00)	0 (0.00)	0 (0.00)	0 (0.00)	0 (0.00)
*Tachyglossus aculeatus*	0 (0.00)	0 (0.00)	0 (0.00)	0 (0.00)	2 (0.02)	0 (0.00)
Invertebrates	51 (0.08)	121 (0.19)	199 (0.18)	133 (0.09)	6 (0.06)	23 (0.07)
Vegetation	133 (0.20)	175 (0.28)	183 (0.17)	232 (0.16)	21 (0.20)	54 (0.17)
Birds	30 (0.04)	31 (0.05)	53 (0.05)	59 (0.04)	2 (0.02)	13 (0.04)
Reptiles	47 (0.07)	75 (0.12)	64 (0.06)	38 (0.03)	1 (0.01)	27 (0.09)

**Figure 4:**
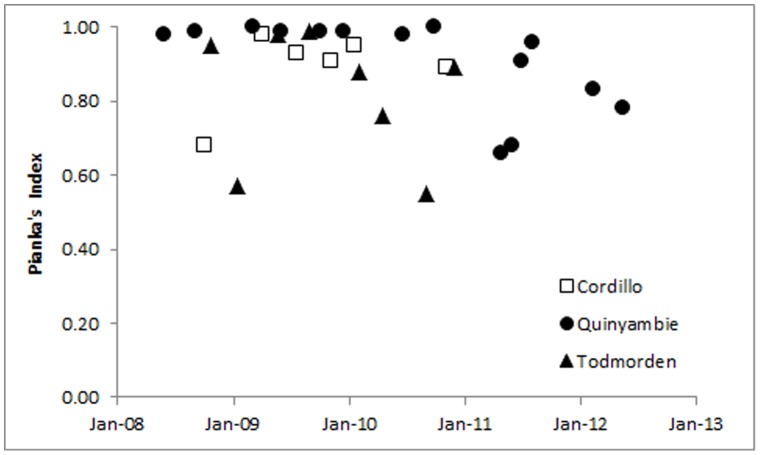
Temporal variation in dingo dietary similarity (*O* = ) between baited and unbaited treatment areas at three sites in northern South Australia, May 2008 to May 2012.

Overall Pianka's index values for Cordillo Downs (*O* = 0.99), Quinyambie (*O* = 1.00) and Todmorden (*O* = 0.96) likewise indicated that dingo diets in baited and unbaited areas were identical or near-identical at each site. Moreover, diets of dingoes in baited and unbaited areas were near-identical for most individual surveys at each site as well (Table 4). Dietary similarity did not change through time for Cordillo Downs (r = 0.4590, df 5, p = 0.3598) or Todmorden (r = −0.2115, df 7, p = 0.6151). At Quinyambie, dingo diets were identical or near-identical for over two years subsequent to the commencement of baiting (r = 0.2402, df 7, p = 0.5667). Dietary similarity between treatments at Quinyambie appeared to decline in the latter half (years 3 and 4) of the study in 2011 and 2012 (r = −0.6474, df 13, p = 0.0123; Fig. 4) subsequent to the landscape-changing effects of the substantial rainfall events which occurred during this period [60]. Importantly however, for all sites and for the survey by survey analysis only (i.e. Fig. 4), Pianka's index values where *O* = <0.85 were typically associated with sample sizes too low for a meaningful comparison of similarity between treatments (Table 4).

## Discussion

Our results demonstrate that lethal dingo control did not alter dingo dietary diversity or similarly at our study sites. Brillouin's index values showed that the diversity of prey consumed by dingoes was near-identical between baited and unbaited areas at each site, indicating that dingoes in baited areas selected neither a wider nor narrower range of prey than dingoes in unbaited areas. Pianka's index values showed that the overall proportion of various prey consumed by dingoes was also either identical or near-identical between baited and unbaited areas; a trend reflected in results for individual surveys as well (Table 4; Fig. 4). This indicates that dingoes in baited and unbaited areas consumed the same proportion of various prey independent of the diversity or suite of prey species available (Table 5, Fig. 2). That temporal trends in the proportion of scats containing primary dingo prey were also similar between baited and unbaited areas further shows that prey consumption by dingoes is independent of dingo control (Fig. 3). These findings were consistent between sites dominated by the availability of large prey (Todmorden), small prey (Quinyambie) or mixed-sized prey (Cordillo Downs), suggesting that these results may be common across different ecosystems with different mammal assemblages.

Corbett and Newsome [25] showed that prey selection by dingoes varies according to the availability of different sizes of prey (from rodents to cattle), where dingoes typically prefer small mammals but consume an increasing proportion of larger mammals as smaller ones become unavailable. Newsome and colleagues [52], [61] later showed that provision of anthropogenic food subsidies (such as livestock and rubbish) can disrupt this pattern, providing an essentially stable or year-round supply of food, which can lead to increased risk of hyperpredation to some threatened prey species (see also [23]). Additional information addressing optimal foraging theory and prey switching indicates that dingoes can easily and rapidly switch between prey of different types (i.e. mammals, birds, invertebrates) or sizes (e.g. [15], [20], [24], [26], [62]), consistent with the energetic studies of carnivores more generally [1], [7]. Lone dingoes are also capable of capturing and subduing adult kangaroos if necessary [26], [63]. These findings suggest that although dingoes can and do switch between various prey types and sizes, perturbations to dingoes' social structure (such as the loss of individuals through baiting) may not automatically cause population-level changes in dingo diets. If dingoes in baited areas switched to prey different to those used by dingoes in nearby unbaited areas as a result of baiting, Pianka's index values should have been different between treatment areas or decreased over time, but such did not occur (Table 4, Fig. 4). If dingoes in baited areas utilized a wider or narrower range of prey than dingoes in nearby unbaited areas as a result of baiting, Brillouin's index values should have been markedly different, but such did not occur either (Fig. 2).

Although lethal control temporarily reduces dingo abundances (Table 2) and undoubtedly alters their social structures to some degree through poisoning or removal of some individuals [29], [38], [60], baiting did not appear to alter population-level dingo diets at our sites. These findings increase our understanding of the potential effects of lethal control on the behaviour and function of social mid-sized carnivores. Flexible social structures and generalist diets likely increase the resilience of such species to fluctuations in resource availability and biotic and abiotic perturbations that might normally be problematic for obligate group-hunting carnivores. Such characteristics likely contribute to the invasion success and global resilience of dingoes, coyotes (*Canis latrans*), red foxes and some other mid-sized carnivores (see [64] for details).

Some have called for cessation of lethal dingo control, claiming that such management approaches negatively affect dingo abundance and function and initiate trophic cascades that lead to biodiversity declines [9], [31], [65], [66]. However, the experimental and empirical results of this study (and many others) do not support such claims. That the national distribution and density of dingoes is naturally increasing despite attempts at broad-scale lethal control [27] suggests that dingo function is not altered by baiting in ways that curtail dingo population growth. That dingo pack structure and social stability is resilient to moderate levels of lethal control [67] suggests that population growth rates are not inhibited by the ongoing removal of some individuals (see also [68]). That dingo movement behaviour and detectability is not always altered by lethal control [35] suggests that dingo function is not altered in ways that might affect their study or their per capita contact rates with sympatric predator or prey species. That dingoes' numerical relationships with other sympatric predators are not altered by lethal control over time [29] suggests that dingo abundance or function is not altered in ways that might increase densities of other predators in extant food webs. That sympatric prey population trends fluctuate independent of dingo control [30] suggests that threatened fauna populations are not harmed by contemporary dingo control practices. That dingoes do not alter their diets in response to lethal control (this study) concurs with each of these previous findings, and further suggests that predation of particular prey types or species is not exacerbated by lethal control, but is rather a function of dingo density and prey/food availability and preference [23], [52], [69].

We conclude that while some large top-predators are clearly reliant on intact or robust group numbers or social structures to secure sufficient prey resources (such as lions, grey wolves or African wild dogs), mid-sized top-predators with flexible and generalist diets (such as dingoes) may be functionally and numerically resilient to repeated moderate levels of population reduction over time in many cases. Where threatened fauna are expected to be influenced by managed predator populations, future studies might investigate the responses of threatened prey populations to lethal predator control (such as [30]) to confirm the potential effects of predator control on lower trophic levels.
